# Celiotomy to collect spermatogonial stem cells in *Brycon orbignyanus* for species preservation

**DOI:** 10.1590/1984-3143-AR2025-0085

**Published:** 2026-03-02

**Authors:** Laís Gonçalves da Silva, Karel Gelina Torres-Lozano, Rômulo Batista Rodrígues, Thaiza Rodrigues de Freitas, Jayme Aparecido Povh, Louise Nex Spica, Nathalia dos Santos Teixeira, Douglas Cosme Selle, Jhony Lisbôa Benato, Raquel Santos dos Santos, Eduardo Thomé Nicoleti, Renata Villar Dantas, Danilo Pedro Streit

**Affiliations:** 1 Grupo de Pesquisa Aquam, Programa de Pós-graduação em Zootecnia, Universidade Federal do Rio Grande do Sul – UFRGS, Porto Alegre, RS, Brasil; 2 Programa de Pós-graduação em Ciência Veterinária, Universidade Federal do Rio Grande do Sul – UFRGS, Porto Alegre, RS, Brasil; 3 Departamento de Zootecnia e Ciências Biológicas, Universidade Federal de Santa Maria – UFSM, Palmeira das Missões, RS, Brasil; 4 Programa de Pós-graduação em Ciência Animal, Faculdade de Medicina Veterinária e Zootecnia – FAMEZ, Universidade Federal Mato Grosso do Sul – UFMS, Campo Grande, MS, Brasil

**Keywords:** endangered species, gonadectomy, SSC, surgery, piracanjuba

## Abstract

Surgical techniques in aquatic species are underdeveloped, despite these species comprising a significant superclass among vertebrates. In the context of species preservation, studies involving the use of reproductive tissues typically involve the euthanasia of donors. Thus, in animals at high risk of extinction, the practice of sacrificing them presents a point of contradiction between ex situ conservation efforts. The objective of this study was to compare the viability of spermatogonia stem cells (SSCs) collected from *Brycon orbignyanus* using conventional methods (euthanasia) versus a surgical procedure. Lateral celiotomy was performed on 27 immatures males to obtain a portion of gonadal tissue. The fish were divided into three groups (n=9), with each group receiving polyglactin 910, polyester, or catgut sutures, respectively, for celiorrhaphy. Dermorraphy was performed using nylon sutures in all groups. A. An additional euthanasia group consisted of nine animals exposed to 20 mg/L of propofol. The survival rate over a 48-hour period was 100% for the polyglactin 910 group and 77.7% for the polyester and catgut groups. Additionally, the viability of SSCs was similar between the euthanasia and surgical procedures. The lateral celiotomy technique is feasible for obtaining SSCs in fish with laterally compressed anatomy, such as *B. orbignyanus*. Additionally, the technique allows the preservation of SSCs as a model for endangered fish species.

## Introduction

The agenda for strategies regarding the conservation and sustainable use of aquatic genetic resources (AqGR) is considered fundamental by the Food and Agriculture Organization (FAO, 2023). Among these conservation strategies, in situ preservation of wild and farmed fish for reproduction and genetic material collection is of utmost importance. Similarly, ex situ conservation emphasizes the need to establish germplasm banks, not only for species bred in captivity but also for wild species (FAO, 2019).

The use of gametes in the establishment of fish germplasm banks can be crucial for preserving the DNA of animals with high zootechnical value (Thelie et al., 2019) or species at risk of extinction (Hagedorn et al., 2018; Swaminathan et al., 2020). Genetic variability within a population and the adaptive genetic traits of a species depends on both the male and female genome. In this context, spermatogonial steam cells (SSCs) emerge as an alternative for preserving genetic diversity, given their potential for transplantation into other fish. Due to their genetic plasticity, SSCs have the ability to differentiate into both oocytes and sperm, depending on the somatic environment in which they develop (Lee et al., 2013; Okutsu et al., 2006).

Among the potential uses of SSCs, animal reproduction, production of transgenic animals and conservation of endangered species are recurrently highlighted applications (Oatley, 2017; Honaramooz and Yang, 2010). In fish, the use of SSCs is almost exclusively related to the use in the transplantation of undifferentiated cells to another recipient fish, which is called a "surrogate mother" (Wylie et al., 2025). The use of SSCs from genetic preservation, allows the supply of reproductive material throughout year-round, facilitating juvenile fish production (Abualreesh et al., 2020) in addition to transplantation into adult fish (Majhi et al., 2020; Xie et al., 2020).

To access SSCs, it is necessary to obtain a fragment of gonadal tissue, which is routinely collected after euthanasia (Hagedorn et al., 2018; Rivers et al., 2020; Rosa et al., 2023). Even when ethical standards are strictly followed, sacrificing the animal poses a challenge to in situ preservation strategies. The growing interest in using fish as experimental models, combined with the development and performance of surgical procedures, has been timidly explored. Surgical techniques commonly used in terrestrial animals can be adapted for fish (Sladky and Clarke, 2016). The first report of gonadectomy via lateral celiotomy dates to the 1960s (Jhons and Liley, 1970). However, there has been a notable absence of reports on surgical techniques to fish with laterally compressed anatomy that does not allow access via the ventral region, such as *Brycon orbignyanus*.

Numerous species of the genus *Brycon* are listed and classified at different levels of threat regarding their wild populations. And this scenario is no different for *B. orbignyanus*, which is classified as a critically endangered species (ICMBio, 2018). The almost complete reduction of wild stocks of the species has been reduced in almost its entirety (Oliveira et al., 2017; Agostinho et al., 2008). And studies have related the need for strategies to protect the species, either in situ (Tonella et al., 2019) or ex situ (Galo et al., 2019). As for the ex-situ strategy, the recent study by Siqueira-Silva et al. (2021) showed viability from the transplantation of SSCs.

The objective of this study was to compare the viability of spermatogonia stem cells (SSCs) collected from *B. orbignyanus* using conventional methods (euthanasia) versus a surgical procedure. Furthermore, it demonstrates the successful development of the protocol in an endangered species (Siqueira-Silva et al., 2021).

## Methods

### Ethics statement

This study was conducted with the established guidelines of the Conselho Nacional de Controle e Experimentação Animal – CONCEA (National Council for Control and Animal Experimentation) and by the approval of the Animal Use Ethics Committee of the Federal University of Rio Grande do Sul (CEUA-UFRGS), project number 44839.

### Fish maintenance and experimental conditions

Thirty-six males *B. orbignyanus*, were randomly selected from CEMIG aquaculture station, located in Itutinga, (Minas Gerais - Brazil). The animals were five years old, with an average weight and length of 452.51*±*101.20 g and 33.91*±*2.48 cm, respectively, and stocked in a breeding maintenance tank at a density of one animal per 3 m^2^.

After selection, the animals were kept in the laboratory in three 1000 L aquariums at 22 °C, with constant water renewal for the experiment.

### Experimental design

#### Experiment 1

**Figure 1:**
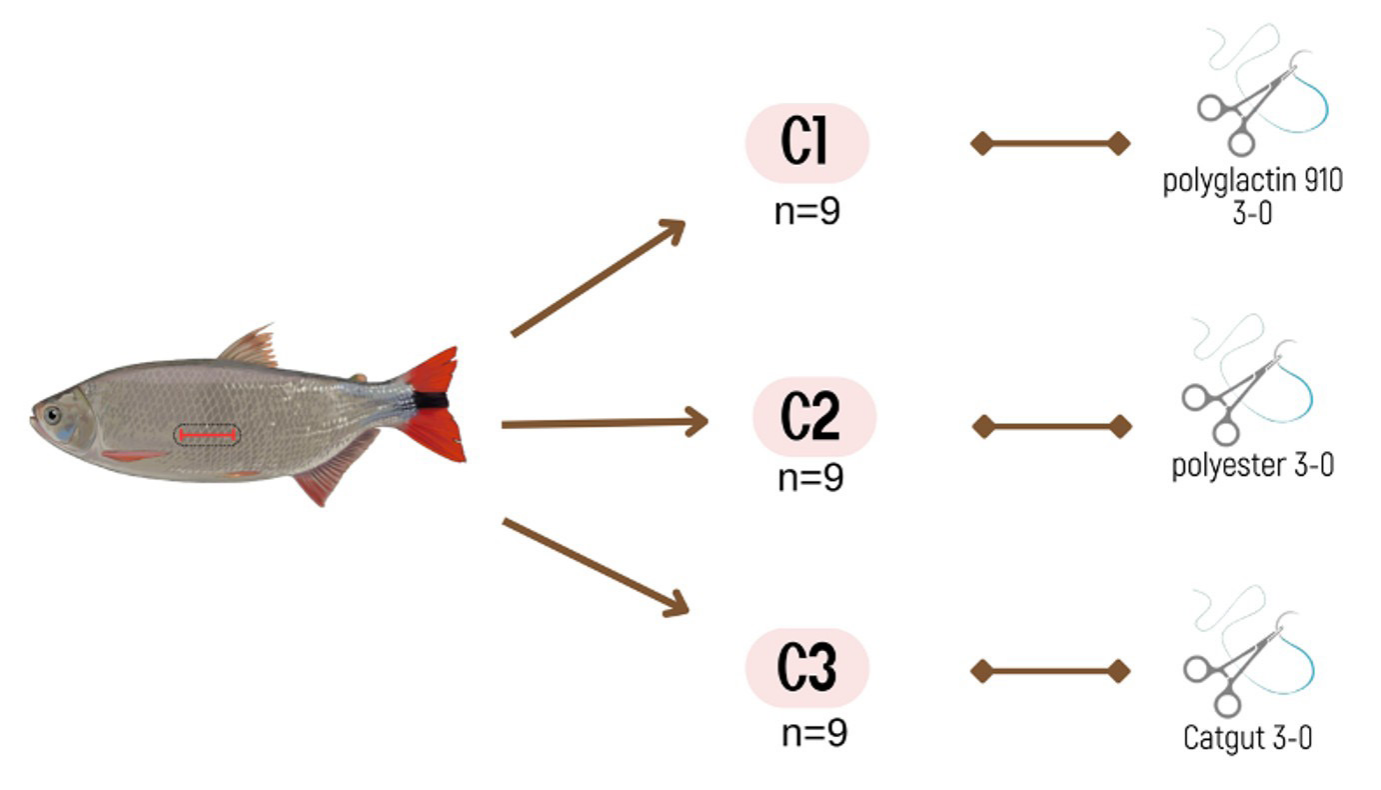
Incision area and treatment groups with the number of *Brycon orbignyanus* and different suture used.

To access testicular tissue and collect spermatogonial stem cells (SSCs) in fish with laterally compressed anatomy, such as piracanjuba, the surgical method described by Jhons and Liley (1970) was used. After collecting SSCs, three types of threads were tested for suturing the *B. orbignyanus* (Figure 1). Thus: C1 - polyglactin 910 3-0, C2-polyester 3-0 and C3- catgut 3-0 sutures for celiorrhaphy after unilateral partial gonadectomy. Nine animals were used in each experimental group.

#### Experiment 2

**Figure 2:**
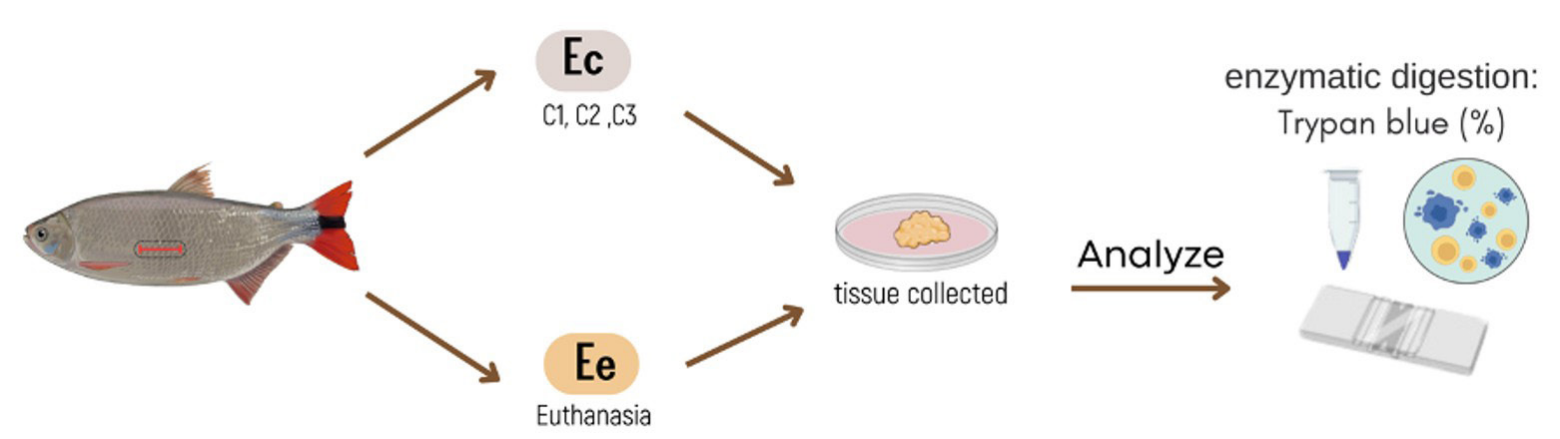
Treatments using surgical procedure with the Euthanasia group (Ec) to collect testicular tissue and the test validation teste for enzymatic digestion by tripan blue staining to analyzed spermatogonial steam cells (SSC) of *Brycon orbignyanus*.

The SSCs that were obtained by surgery in experiment 1 (Ec) were compared with the SSCs obtained by the conventional method, euthanasia (Ee) (Figure 2).

### Anesthesia

The fish were collected from the aquariums and transferred to an induction tank with 12 L of aquarium water mixed with propofol (Propovan, Cristália-São Paulo) at a concentration of 2.5mg/L (Oda et al., 2014). Behaviors such as longitudinal axis rotation, loss of the escape reflex to fin pinching, and reduced opercular movements were monitored to ensure the correct anesthetic depth for surgery (Schottger and Julin, 1967). After induction, the fish’s length (cm) and weight (g) were measured.

The fish were then transferred to a 4 L aquarium containing 1.5 mg/L propofol for anesthetic maintenance. The animals were then positioned in right lateral decubitus position on a surgical bed made of foam covered with cotton fabric, with a silicone hose attached to an aquarium pump delivering 220 L/h of water into the oral cavity for anesthetic maintenance. Dissolved oxygen (5.5 mg/L) was supplied, and water circulated through the opercula. The animal’s eyes were covered with wet tissue to protect the ocular surface, and the body was draped with a sterile, non-woven surgical drape (Polarfix - São Paulo) to maintain moisture during the procedure. Before surgery, 0.5 mg/kg of morphine, 7 mg/kg of enrofloxacin, and 2.2 mg/kg of flunixin were administered intramuscularly (Martin et al., 2021) in the lateral region near the dorsal fin.

### Surgical procedure

**Figure 3:**
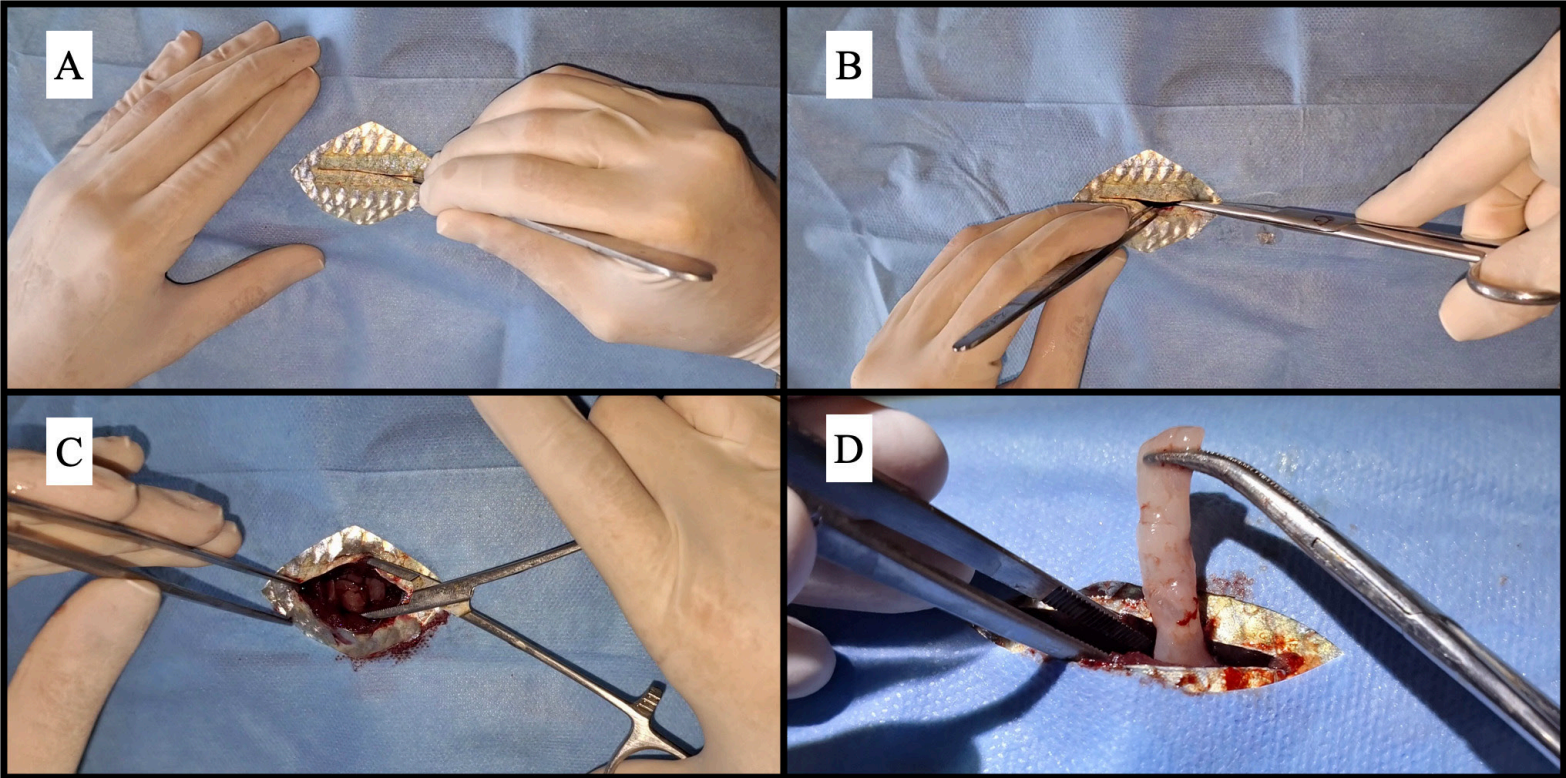
(A) Scalpel blade incision; (B) Ribs section with blunt-tipped scissors; (C) Opening of the coelomic cavity; (D) Excision of the fragment of the gonad of *Brycon orbignyanus* to obtain spermatogonial cells.

The area was prepared using a surgical drape, and scales were removed in a 1 cm by 1 cm area along the lateral midline, from the posterior part of the pectoral fin to one-third of the total pelvic fin length. The area was disinfected with 0.92% saline solution. An incision was made with a #15 scalpel blade, extending approximately 5 cm in a cranio-caudal direction, 0.5 cm below the lateral midline (Figure 3A). Muscular tissue was separated using Met- zenbaum scissors, and the ribs (3^rd^ and 4^th^) were sectioned with blunt-tipped scissors to access the coelomic cavity (Figure 3B). The left gonad, located in the mid-cranial portion (Figure 3C), was partially excised (0.5 cm fragment) for SSC extraction (Figure 3D).

Celiorrhaphy was performed using a simple continuous suture to approximate the muscle with the materials specific to each treatment group (C1, C2, or C3), followed by dermorrhaphy using a Sultan suture pattern with non-absorbable 3-0 nylon in all groups.

### Surgical recovery

After surgery for C1, C2 and C3 animals, each fish was placed in a recovery tank containing 20 L of water to monitor the complete recovery from anesthesia, confirmed by coordinated swimming. After recovery, the fish were transferred to a tank with 1% saline solution for five minutes (Tavares-Dias, 2022). The animals were then transferred to experimental tanks to monitor the healing of surgical wounds for 48 hours, as well as to observe possible mortalities. After this time, all animals were returned to the breeding tank to reduce the stress of confinement and minimize mortality. Over the course of another 120 hours, the presence of dead animals was observed in this breeding tank.

### Euthanasia

The fish in group Ec were euthanized by immersion in a propofol solution at 20 mg/L, followed by spinal cord section after opercular movements ceased (Balko et al., 2018).

### Validation analyzes for SSCs

The SSC collection followed the protocols of Abualreesh et al. (2020) and Franěk et al. (2019), with modifications. Gonadal fragments from each group were placed in 15 mL falcon tubes with 10 mL Leibovitz L-15 medium (Sigma-Aldrich-USA) pH 7.8 to maintain hydration. They were exposed to a 0.5% hypochlorite solution for two minutes, washed three times with L-15, dried, weighed, and sectioned into 3 × 3 mm fragments. Three fragments were placed in 2.0 mL microtubes and cut into smaller pieces with scissors.

For enzymatic digestion, 50 μL of 0.25 mg/mL trypsin (Dinâmica Química-Brazil) + 0.02 mg/mL EDTA (Neon comercial-São Paulo) was added to the microtubes and incubated on a shaking platform for 1.30 h. To stop enzyme activity, 400 μL of L-15 and 100 μL of fetal bovine serum (FBS) were added. The solution was filtered through a 40 μm nylon filter into 1.5 mL microtubes and centrifuged at 500G for 20 minutes. The supernatant was discarded, and the cell pellet was resuspended in 300 μL of L-15 + 10% FBS.

Membrane integrity of the spermatogonial cells was assessed according to Franěk et al. (2019). A mixture of 10 μL of the cell suspension and 10 μL of 0.4% trypan blue was placed in a Neubauer hemocytometer. Five fields of the hemocytometer were analyzed under a microscope (Nikon E200, Tokyo, Japan) at 40X magnification, and live cells were counted in triplicate.

### Statistical analysis

Data were analyzed using normality tests (Shapiro-Wilk and Kolmogorov-Smirnov) and homogeneity tests (Bartlett). Parametric data were analyzed by one-way analysis of variance (One-Way ANOVA), and when sig- nificant differences were observed, means were compared using Tukey’s test. Parametric data are presented as bar graphs (mean *±* standard deviation). Non-parametric data were analyzed using the Kruskal-Walli’s test, and differences among groups were tested by Dunn’s test. Non-parametric data are presented in Box-Whisker plots (mean, minimum, and maximum values). Analyses and graph creation were performed using GraphPad Prism 9.0 software.

## Results

### Experiment 1

**Figure 4:**
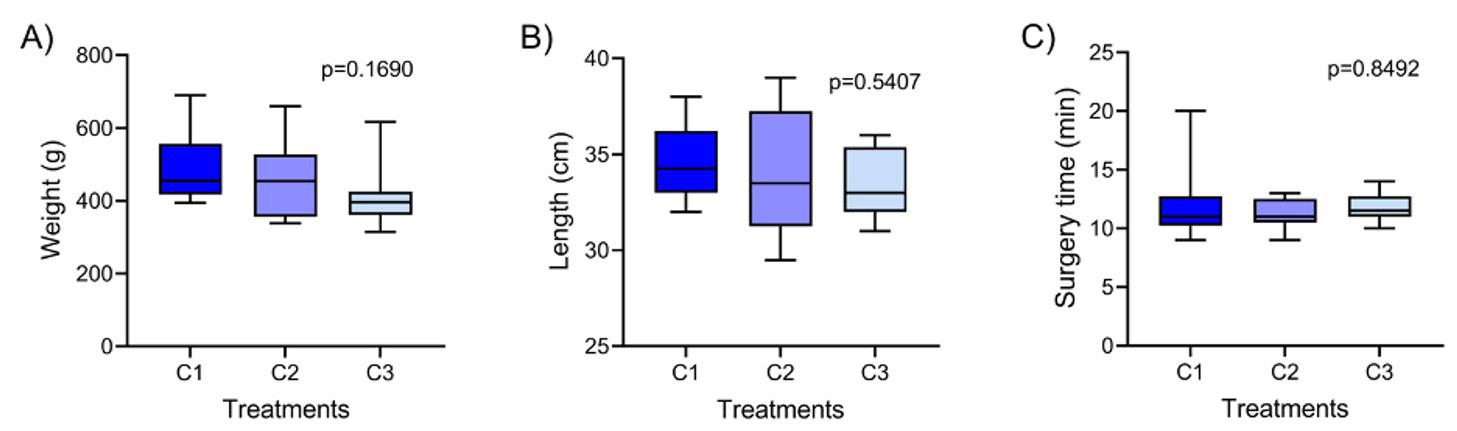
Characteristics of *Brycon orbignyanus* and surgery time. (A) Weight (g) (p=0.1690); (B) Total length (cm) (p=0.5407); (C) Surgery time (min) (p=0.8492). No differences were observed between the experimental groups according to the Kruskal-Walli’s test.

There was no significant difference regarding the weight and length of the animals that made up the experimental groups, as well as for the surgery time using the different types of threads tested for suturing the animals (Figure 4A-C).

**Figure 5:**
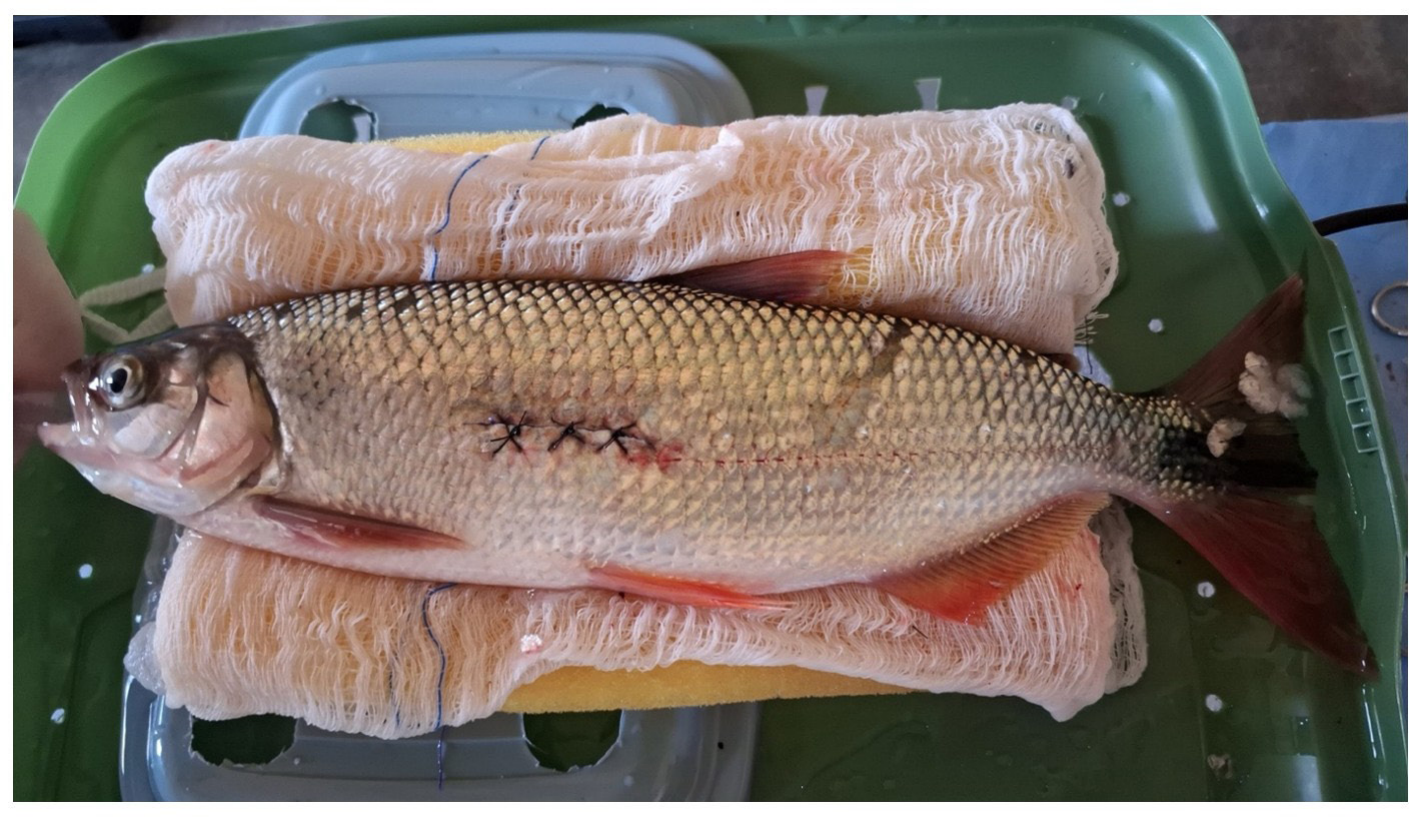
Immediate postoperative period of a *Brycon orbignyanus* specimen.

In a descriptive analysis, there was no difference between the groups in the immediate postoperative period concerning the extent of the surgical wound for strategic access to the gonad. All groups showed ease in locating the target tissue, minimal muscle wall bleeding during the procedure, and satisfactory edge coaptation (Figure 5).

**Figure 6:**
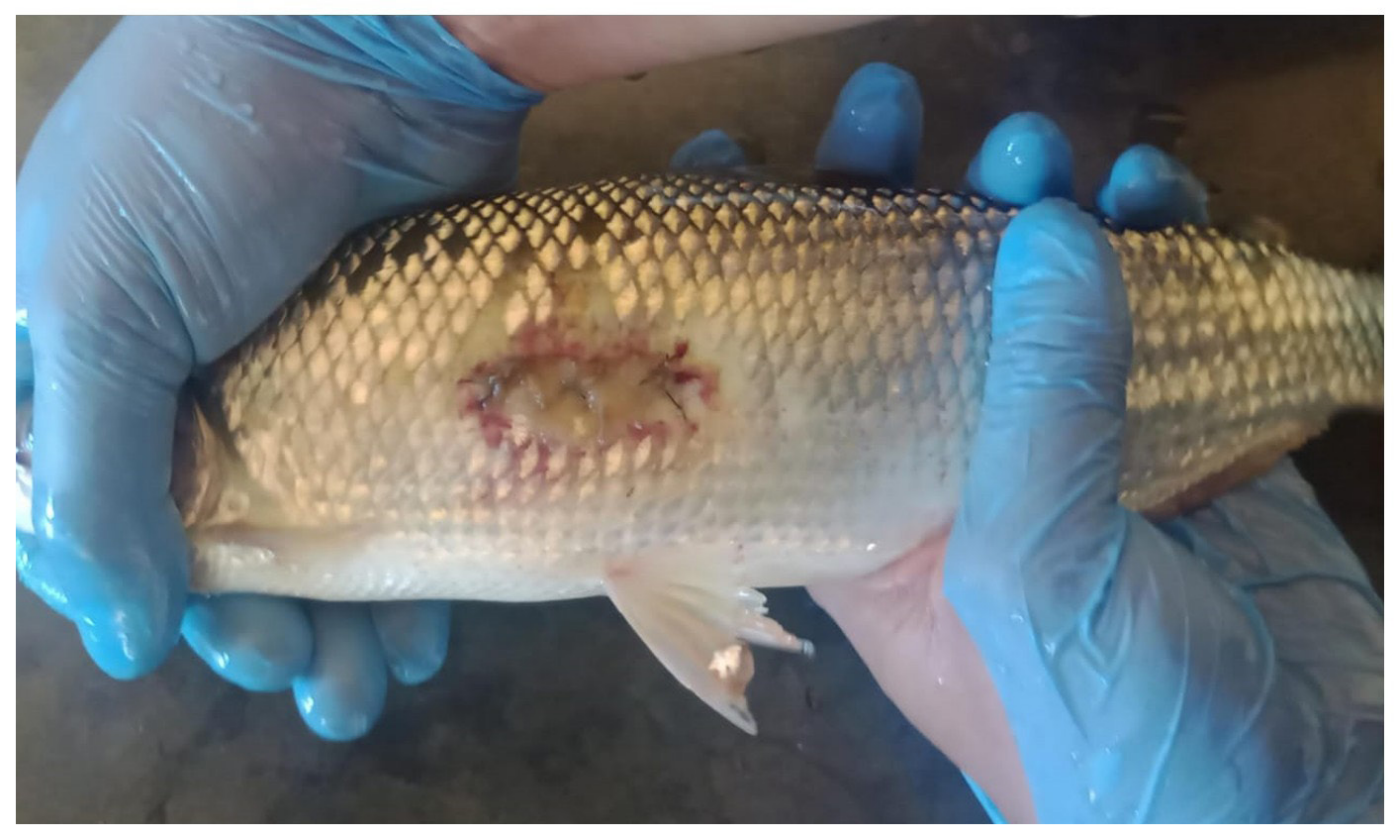
Presence of a yellowish gelatinous structure over the surgical wound, observed 36 hours postoperatively in *Brycon orbignyanus*.

After 36 hours of postoperative monitoring, gelatinous structures with a cotton-like appearance when immersed in water and a yellowish coloration were observed on the fins, eyes, oral cavity, and surgical site. Despite the presence of these structures over the surgical incision site, none of the animals showed suture dehiscence or wound opening during the observation period (Figure 6).

**Figure 7:**
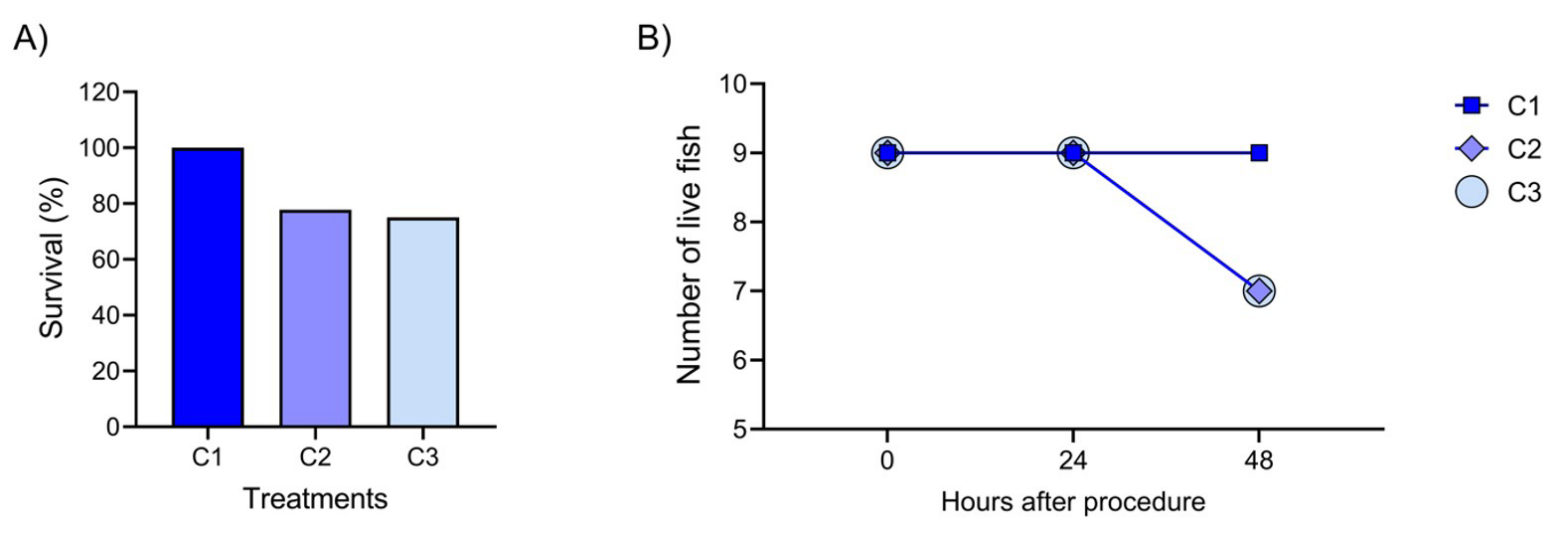
(A) Survival demonstration 48 hours after surgery in *Brycon orbignyanus*; (B) Mortality rate of the groups after the surgical procedure for SSC collection during the observation period.

Survival was recorded up to 48 hours after surgery across the groups (Figure 7A). During the first 36 hours, no deaths were recorded in any group. At the 48-hour evaluation, groups C2 and C3 showed a mortality rate of 22.2% (n=2) each (Figure 7B).

### Experiment 2

**Figure 8:**
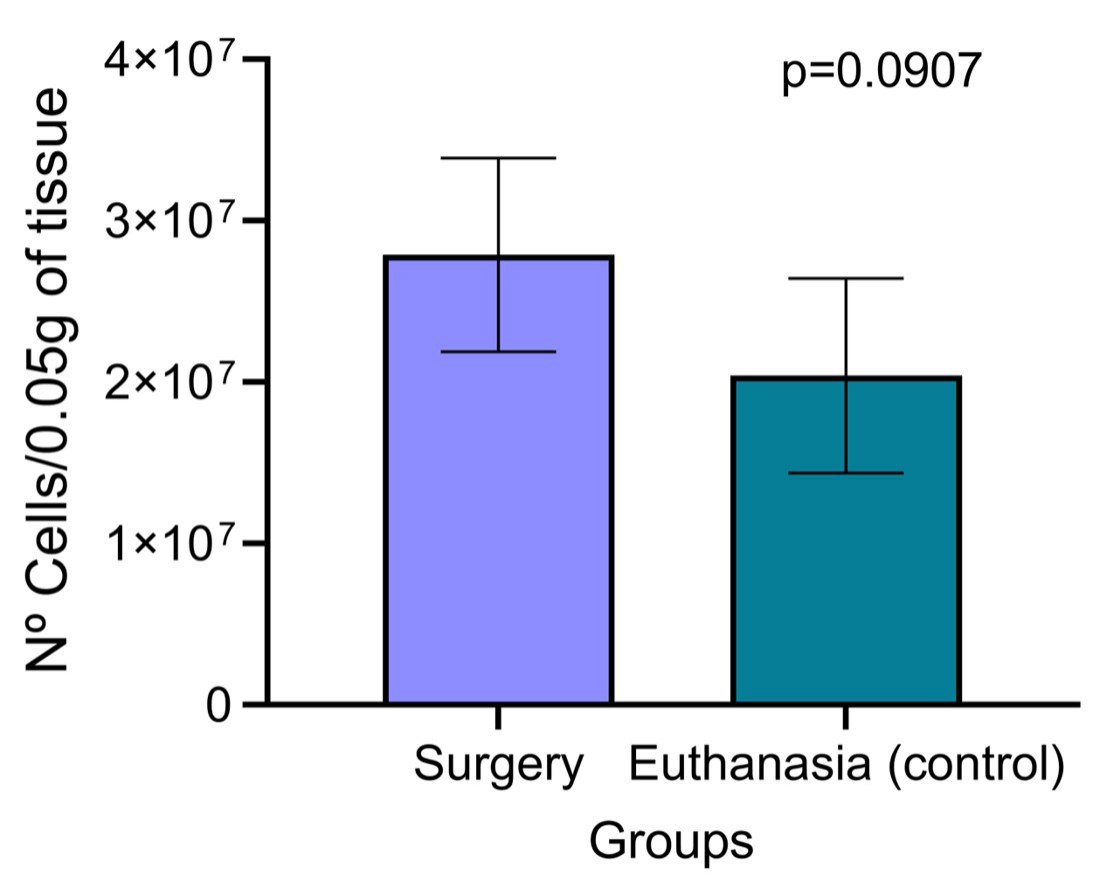
Comparison among the number of cells corrected for 0.05 grams of tissue of *Brycon orbignyanus*, presented in bars. The surgery group is represented in light blue, and the euthanasia group (control) in dark blue.

When comparing viability among spermatogonia obtained by the surgical method and the euthanasia method, no statistical difference was observed between the methods, as shown in Figure 8.

## Discussion

The efficacy of lateral celiotomy for unilateral partial gonadectomy presented in this study, brings a novel alternative for collection of spermatogonial steam cell, aiming the conservation of an endangered fish species. However, surgical procedures in species with compressed anatomy is challenged, due the difficult to have access into the celomatic cavity.

The wide diversity of particularities among species is possibly the main challenge for those managing these animals, whether for aquariums, production, or conservation purposes (Zaniboni Filho and Ribolli, 2024; Tiersch and Yang, 2019). The initial challenge imposed in the development of the surgical technique that emerged for spermatogonia collection was the laterally compressed anatomy of *B. orbignyanus*. Studies describing the use of surgery in fish are scarce and, with the exception of Jhons and Liley (1970), the surgical access incision is always recommended in the ventral region of the animal. This can be observed in the studies of Brown and Richards (1979) (*Salmo salar and Salmo trutta*), Chapman and Park (2005) (*Acipenser oxyrinchus de sotoi*) and Majhi et al. (2020) (*Clarias magur*). Even in books (McLean, 1994; Johnson, 2000) or reviews (Wildgoose 2000) that address surgery in fish, the suggested access is always ventral. It is worth noting that the species related in these studies have a fusiform anatomy, that is, a flattening of the ventral region, facilitating surgical access.

The laterally compressed anatomy of *B. orbignyanus* presents significant difficulty in accessing the celomatic cavity due to the ventral junction of the animal flattened sides. The lateral access method described by Jhons and Liley (1970) for *Trichogaster trichopterus* was used to facilitate the localization of SCC’s, which are in the most dorsal portion of the coelomic cavity in these animals, ensuring safety in the application of the gonadectomy technique. This is consistent with the premise of surgical procedures in terrestrial animals, as noted by Sladky and Clarke (2016), which must be adapted to the species and utilize materials commonly used in planned surgeries.

The time required for the gonadectomy procedure via lateral access applied to *B. orbignyanus* was another relevant point in the development of the methodology proposed in this study. It is important to understand that this is an aquatic animal and necessarily requires an adapted structure to survive outside its comfortable environment. The surgical procedure took between 11-12 minutes, regardless of the suture used, which is comparable to the time reported by Majhi et al. (2020) for *C. Magnum* using ventral access. Additionally, in *S. salar* and *O. mykiss,* the average time was 15 minutes, also using ventral access (McLean 1994).

After the surgical procedure, there was considerable consistency in the approximation of the incision sites across the different sutures tested. The polyglactin 910 3-0 suture used in the C1 group was also employed by Chapman and Park (2005) and was associated with good surgical outcome. On the other hand, Majhi et al. (2020) reported great efficiency using catgut 3-0 sutures for sperm collect in *Clarias magnum,* achieving complete healing within four weeks.

None of the animals exhibited external suture openings with the nylon 3-0 suture during the 48 hours observation period, indicating an effective healing process across all groups. This finding is comparable to the study by Bockelmann et al. (2010), which involved amputating the caudal fin of *Cyprinus carpio*. In that study, surgical wound healing occurred with the formation of an apical epidermal layer within the first two postoperative days.

In fish, stress is influenced by environmental and physical factors, including management, dominance, crowding, aggression, and water quality. These factors lead to immune suppression, which directly affects fish survival (Tort, 2011). *B. orbignyanus* is a sensitive species, and like many others, they are negatively affected by confinement and show improvement once released (Johnson, 2000). During the 48-hour postoperative observation period, the C1 group showed 100% survival rate, while the C2 and C3 groups showed a 77.7% survival rate, respectively. Once relocated to breeding tank no additional deaths were reported.

Germplasm banks are a strategic tool for the conservation of *ex situ* genetic resources. Cryopreservation of *SSCs* has the potential to save species anywhere, as spermatogonia plasticity is considered a valuable tool in combating species extinction (Hagedorn et al., 2018). Commonly, protocols for collecting spermatogonial cells in different species for cryopreservation are developed with the euthanasia of the animals, as described for *Asterropteryx semipunctata* (Hagedorn et al., 2018)*, Melanotaenia fluviatilis* (Rivers et al., 2020) and *Rhamdia quelen* (Rosa et al., 2023). In our study, we observed no difference in the number of viable *SSCs* collected from animals undergoing surgery compared to those collected through euthanasia, indicating that surgical procedures can be a viable alternative for obtaining these cells without sacrificing the animals.

In this study, for the first time an efficient method of lateral celiotomy to collect SSCs in a fish species with a laterally compressed anatomy. This technique can be replicated in other species with the same anatomical structure, thereby preserving the life of the fish, which often needs to be sacrificed for SSC collection. It is important to emphasize that *B. orbignyanus* is a species classified as critically endangered and urgently requires effective conservation strategies. In this sense, greater care must be taken with each of these specimens, even though their role as SSC donors is preponderant to, for example, recover threatened populations from gene banks.

## Conclusion

In conclusion, lateral celiotomy was efficient in collecting SSCs from a species with laterally compressed anatomy, avoiding the sacrifice of *B. orbignyanus*, classified as endangered.
